# Sensitivity of transbronchial lung cryobiopsy in the diagnosis of different interstitial lung diseases

**DOI:** 10.1038/s41598-022-26510-6

**Published:** 2022-12-21

**Authors:** Yoshiaki Zaizen, Yuri Tachibana, Mutsumi Ozasa, Yasuhiko Yamano, Reoto Takei, Yasuo Kohashi, Kensuke Kataoka, Yuji Saito, Kazuhiro Tabata, Masaki Okamoto, Masaki Tominaga, Kiminori Fujimoto, Noriho Sakamoto, Kazuto Ashizawa, Hiroshi Mukae, Andrey Bychkov, Takeshi Johkoh, Tomoaki Hoshino, Yasuhiro Kondoh, Junya Fukuoka

**Affiliations:** 1 Department of Pathology, Nagasaki University Graduate School of Biomedical Sciences, 1-7-1 Sakamoto, Nagasaki, 852-8501 Japan; 2 Division of Respirology, Neurology and Rheumatology, Department of Medicine, Kurume University School of Medicine, 67 Asahi-Machi, Kurume, Fukuoka 830-0011 Japan; 3 Department of Pathology, Kameda Medical Center, 929 Higashi-Cho, Kamogawa, Chiba 296-8602 Japan; 4 Department of Respiratory Medicine, Nagasaki University Graduate School of Biomedical Sciences, 1-7-1 Sakamoto, Nagasaki, 852-8501 Japan; 5 Department of Respiratory Medicine and Allergy, Tosei General Hospital, 160 Nishioiwake, Seto, Aichi 489-8642 Japan; 6 Department of Respiratory Medicine, HARUHI Respiratory Medical Hospital, 8-1 Haruhinagare, Kiyosu, Aichi 452-0962 Japan; 7 Department of Pathology, Kagoshima University Graduate School of Medical and Dental Sciences, 8-35-1 Sakuragaoka, Kagoshima, 890-8544 Japan; 8 Department of Radiology, Kurume University School of Medicine, 67 Asahi-Machi, Kurume, Fukuoka 830-0011 Japan; 9 Department of Clinical Oncology, Nagasaki University Graduate School of Biomedical Sciences, 1-7-1 Sakamoto, Nagasaki, 852-8501 Japan; 10 Department of Radiology, Kansai Rosai Hospital, 3-1-69 Inabasou, Amagasaki, Hyogo 660-8511 Japan

**Keywords:** Respiratory tract diseases, Diagnosis, Pathology

## Abstract

The accuracy of transbronchial lung cryobiopsy (TBLC) in each disease for pathological and multidisciplinary discussion (MDD) diagnosis is not yet established. Method: We investigated 431 patients who were classified by MDD diagnosis and were grouped into the disease categories. For each category or disease, we used TBLC samples to calculate the sensitivities of the pathological diagnosis compared with MDD diagnoses. Further, we compared these sensitivities to pathological diagnoses with all clinical/radiological information. Result: The sensitivity for diagnosing idiopathic interstitial pneumonia (IIPs) with TBLC was higher than connective tissue disease associated ILD (CTD-ILD). Idiopathic nonspecific interstitial pneumonia (iNSIP), fibrotic hypersensitivity pneumonitis, and some CTD-ILDs were diagnosed with lower sensitivities compared to IPF. The sensitivity of pathological diagnosis with all clinical/radiological information in IPF was higher than in iNSIP, but not significantly different from other diseases. The overall sensitivity of the pathological diagnosis with clinical/radiological information was 69.0%, significantly higher than without clinical/radiological information. Conclusion: The sensitivity of pathological diagnosis with TBLC was low for some diseases except IPF. The addition of all clinical/radiological information increased the sensitivity of pathology diagnosis by TBLC, which was no less sensitive than IPF for all diseases except iNSIP.

## Introduction

Transbronchial lung cryobiopsy (TBLC) is a widely used method for the pathological diagnosis of interstitial lung disease (ILD)^1^, with a diagnostic yield as high as 80%^2–6^. However, its accuracy for pathological and multidisciplinary discussion (MDD) diagnosis is not yet as accepted as surgical lung biopsy (SLB). Romagnoli et al. reported that the diagnostic concordance rate between TBLC and SLB was as low as 38%^7^. This is in contrast to the concordance rate of 69.2% reported by the COLDICE prospective study^8^, where it was also observed that the diagnostic concordance between TBLC and SLB was high in idiopathic pulmonary fibrosis (IPF), and low in hypersensitivity pneumonitis (HP)^8,9^.

In our study, we compared the diagnostic sensitivity of TBLC in different ILDs to investigate which diseases TBLC can diagnose with high accuracy. We found that usual interstitial pneumonia (UIP) was evaluated with high accuracy, while findings for other ILDs showed more variance^10^. These suggest that the diagnostic sensitivity of TBLC differs depending on the ILD entity.

## Methods

**Figure 1:**
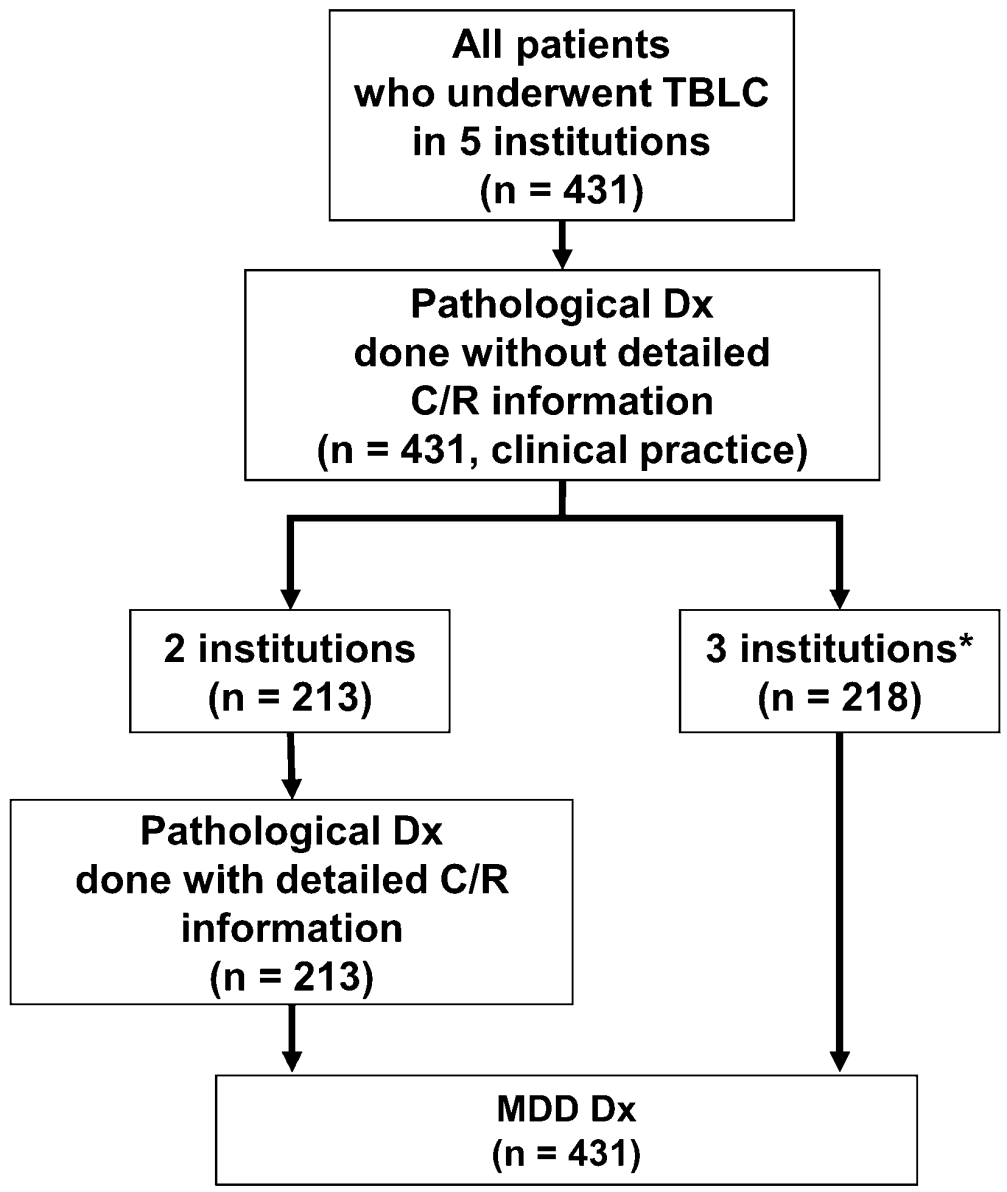
The flowchart on pathology and multidisciplinary discussion (MDD) diagnoses collected for this study. * At these three facilities, pathological diagnosis was performed with only the following information presented: age, sex, and location of the sampling site.

We included all patients who underwent TBLC in five institutions for suspected ILD between January 2018 and June 2021. All patients had routine pathological and MDD diagnoses. MDD diagnosis was performed using TBLC; and we used this as gold standard to examine the sensitivity of the pathology diagnosis with TBLC alone. In the two out of five institutions, pathological diagnoses were routinely made without access to detailed clinical or radiological data (diagnosis without clinical/radiological information); these data were further retrieved, analyzed, and pathological diagnoses were amended accordingly (diagnosis with clinical/radiological information). We also collected clinical information, details in the pathology report, and MDD diagnosis of these cases. MDD was performed using the clinical and radiological information at the time the TBLC was performed. Figure 1 shows a flowchart of the diagnoses we collected in this study.

In the participating institutions, a flexible BF-1T260 or BF-1TQ290 bronchoscope (Olympus Corporation, Tokyo, Japan) and a 1.9-mm or 2.4-mm cryoprobe (Erbe Elektromedizin, Tübingen, Germany) were used for TBLC. All patients underwent intubation with a flexible endotracheal tube and maintained spontaneous respiration under midazolam and fentanyl sedation. The cryoprobe was inserted through the working channel of the flexible bronchoscope, placed under the pleura 1–3 cm away from the pleura under fluoroscopy, and activated for 6–7 s with the 1.9 mm probe and 4–5 s with the 2.4 mm probe.Patients were classified by disease type as per MDD diagnosis and were grouped into the following six categories: idiopathic interstitial pneumonia (IIP), connective tissue disease associated ILD (CTD-ILD), in hypersensitivity pneumonitis (HP), iatrogenic lung disease, unclassifiable ILD, and Other^2,11–13^. For each category, we used TBLC samples to calculate and compare the sensitivities of the pathology-only and MDD diagnoses; to determine if TBLC exhibited relatively high or low diagnostic sensitivity for each disease type. Further, we compared these sensitivities to that of pathological diagnoses done without clinical/radiological information, to investigate whether the additional information improved diagnostic sensitivity in each disease category. We also compared the sensitivity of pathological diagnosis between each category against IIP and IPF using Fisher’s exact test. Statistical significance was defined as *p* < 0.05; all statistical analyses were performed using JMP 16.0 (SAS Institute, Cary, NC, USA).

### Ethical approval

Informed consent was obtained in the form of opt-out methodology between the date of certification and Dec 31, 2022. This study was conducted in accordance with the tenets of the Declaration of Helsinki and approved by the Nagasaki University Hospital Clinical Research Ethics Committee (No. 21081607).

## Results

There were 431 patients included in the study, with a median age of 69 (interquartile range = 60–75) years; 264 (61.3%) patients were male. 50 cases (11.6%) had inadequate sampling (e.g. no specimen or only tiny specimens of < 4 mm, with no alveoli, etc.). Through MDD, 146 (33.9%) patients were diagnosed with IIPs, 88 (20.4%) with HP, and 54 (12.5%) with CTD-ILD. 72 patients (16.7%) who were not diagnosed by MDD with TBLC were categorized as unclassifiable ILD. IPF was diagnosed in 106 patients (24.6%).

Table 1 shows the sensitivity of the pathological diagnosis alone versus diagnosis by MDD for each disease category. Using TBLC, the sensitivity for diagnosing IIP was 65.1%, which was higher than CTD-ILD (*p* = 0.010). The overall sensitivity of pathological diagnosis was 53.6% (231/431), while for IPF, itwas 72.6%. Idiopathic nonspecific interstitial pneumonia (iNSIP) (0.0%, *p* < 0.001), fibrotic HP (50.8%, *p* = 0.009), rheumatoid arthritis associated ILD (RA-ILD) (47.6%, *p* = 0.038), and systemic sclerosis associated ILD (SSc-ILD) (25.0%, *p* = 0.010) were diagnosed with lower sensitivities compared to IPF.

**Table 1:** Diagnostic sensitivity of TBLC for each disease.

MDD diagnosis	Sensitivity without C/R (n = 431)		Sensitivity with C/R (n = 213)	
	n (%)	*P* value	n (%)	*P* value
IIP	95/146 (65.1%)		50/62 (80.7%)	
IPF	77/106 (72.6%)		43/50 (86.0%)	
Idiopathic NSIP	0/6 (0.0%)	< 0.001*	0/2 (0.0%)	0.027*
COP	7/12 (58.3%)	0.323*	1/1 (100%)	1.000*
SR-ILD	7/17 (41.2%)	0.022*	4/7 (57.1%)	0.095*
HP	50/88 (56.8%)	0.215**	42/52 (80.8%)	1.000**
Fibrotic HP	34/67 (50.8%)	0.009*	30/37 (81.1%)	0.567*
Non-fibrotic HP	16/21 (76.2%)	1.000*	12/15 (80.0%)	0.685*
Iatrogenic	9/14 (64.3%)	1.000**	2/5 (40.0%)	0.070**
Drug induced ILD	9/12 (75.0%)	1.000*	2/4 (50.0%)	0.125*
Radiation pneumonitis	0/2 (0.0%)	0.081*	0/1 (0.0%)	0.157*
CTD-ILD	24/54 (44.4%)	0.010**	29/33 (87.9%)	0.566**
RA	10/21 (47.6%)	0.038*	11/12 (91.7%)	1.000*
Sjogren syndrome	2/8 (25.0%)	0.010*	4/5 (80.0%)	0.559*
SSc	2/8 (25.0%)	0.010*	5/6 (83.3%)	1.000*
PM/DM	4/6 (66.7%)	0.668*	5/5 (100%)	1.000*
AAV	3/8 (37.5%)	0.050*	2/3 (66.7%)	0.394*
Other disease	39/57 (68.4%)	0.742**	15/17 (88.2%)	0.722**
Sarcoidosis	8/11 (72.7%)	1.000*	3/4 (75.0%)	0.484*
Infection	6/10 (60.0%)	0.467*	0/1 (0.0%)	0.157*
Pneumoconiosis	2/4 (50.0%)	0.315*	1/1 (100%)	1.000*
Unclassifiable ILD	14/72 (19.4%)	< 0.001**	**9/35(20.5%)**	< 0.001**

Significant values are in bold.

AAV, anti-neutrophil cytoplasmic antibody associated vasculitis; COP, cryptogenic organizing pneumonia; C/R, clinical/radiological information; CTD, connective tissue disease; HP, hypersensitivity pneumonitis; IIP, idiopathic interstitial pneumonia; ILD, interstitial lung disease; IPF, idiopathic pulmonary fibrosis; NSIP, non-specific interstitial pneumonia; PM/DM, polymyiositis/dermatomyositis; RA, rheumatoid arthritis; SR-ILD, smoking related interstitial lung disease; SSc, systemic sclerosis.

* Compared with IPF.

** Compared with IIPs.

The pathological diagnoses of 213 out of 431 patients were evaluated with and without reference to clinical details, blood test results, bronchoalveolar lavage fluid test results, chest radiographs and high-resolution computed tomography (HRCT), which were reviewed using MDD. The sensitivity of pathological diagnosis with clinical/radiological information was 80.7% in IIPs, with no significant difference in the sensitivity between HP, CTD-ILD, and IIPs. The sensitivity of pathological diagnosis with clinical/radiological information for IPF was 86.0%, which was higher than for iNSIP (0.0%, *p* = 0.027). In contrast to the diagnoses without clinical/radiological information, there was no significant difference in pathological diagnostic sensitivity between IPF and fibrotic HP, RA-ILD, and SSc-ILD. The overall sensitivity of the pathological diagnosis with clinical/radiological information was 69.0% (147/213), significantly higher than the cases without clinical/radiological information (111/213, 52.1%, *p* < 0.001).

**Figure 2:**
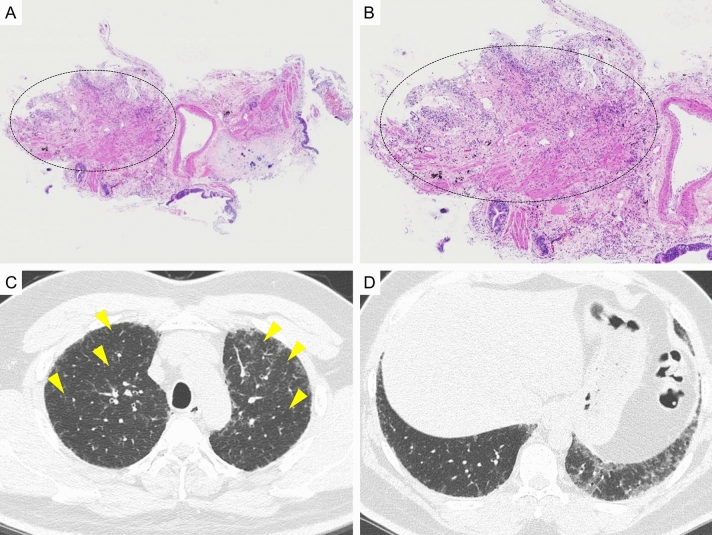
A case in which the pathology diagnosis by transbronchial lung cryobiopsy (TBLC) was inconsistent with the diagnosis of MDD. (**A**), (**B**): pathology by TBLC. Dense fibrosis with architectural destruction were observed (blue curcle). However, normal alveolar structure beside the dense fibrosis was not observed in this specimen. (**C**), (**D**): high-resolution computed tomography (HRCT). Centrilobular nodules were observed in the upper lobes of the lung (orange arrow). In addition, reticular shadows or honeycombing which suggests UIP pattern were not observed.

We present examples of the case in which the diagnosis was discordant between pathology by TBLC and the MDD diagnosis in the current study. In the first case (Fig. 2), pathology by TBLC showed dense fibrosis located in the peripheral of secondary lobules, with smooth muscle hyperplasia and architectural destruction (Fig. 2A,B). These pathological findings were suggestive of UIP, and we diagnosed this case with probable UIP pattern pathologically. However, this pathology specimen did not include normal alveolar structure, which are inherently adjacent to dense fibrosis. The HRCT of this case (Fig. 2C,D) showed small centrilobular nodules in the upper lobes of the lungs. In addition, there were no reticular shadows or honeycombing in the lower lobe of the lung, findings strongly suggestive of UIP. The clinical information of this patient confirmed that anti-pigeon antibodies in the serum were strongly positive. Based on these information, we concluded that HP could not be ruled out, although the TBLC in this case did not identify airway disease in the center of the lobule. Our pathology diagnosis with the clinical/radiological information was consistent with fibrotic HP (Indeterminate for fibrotic HP in the guideline^12^).

**Figure 3:**
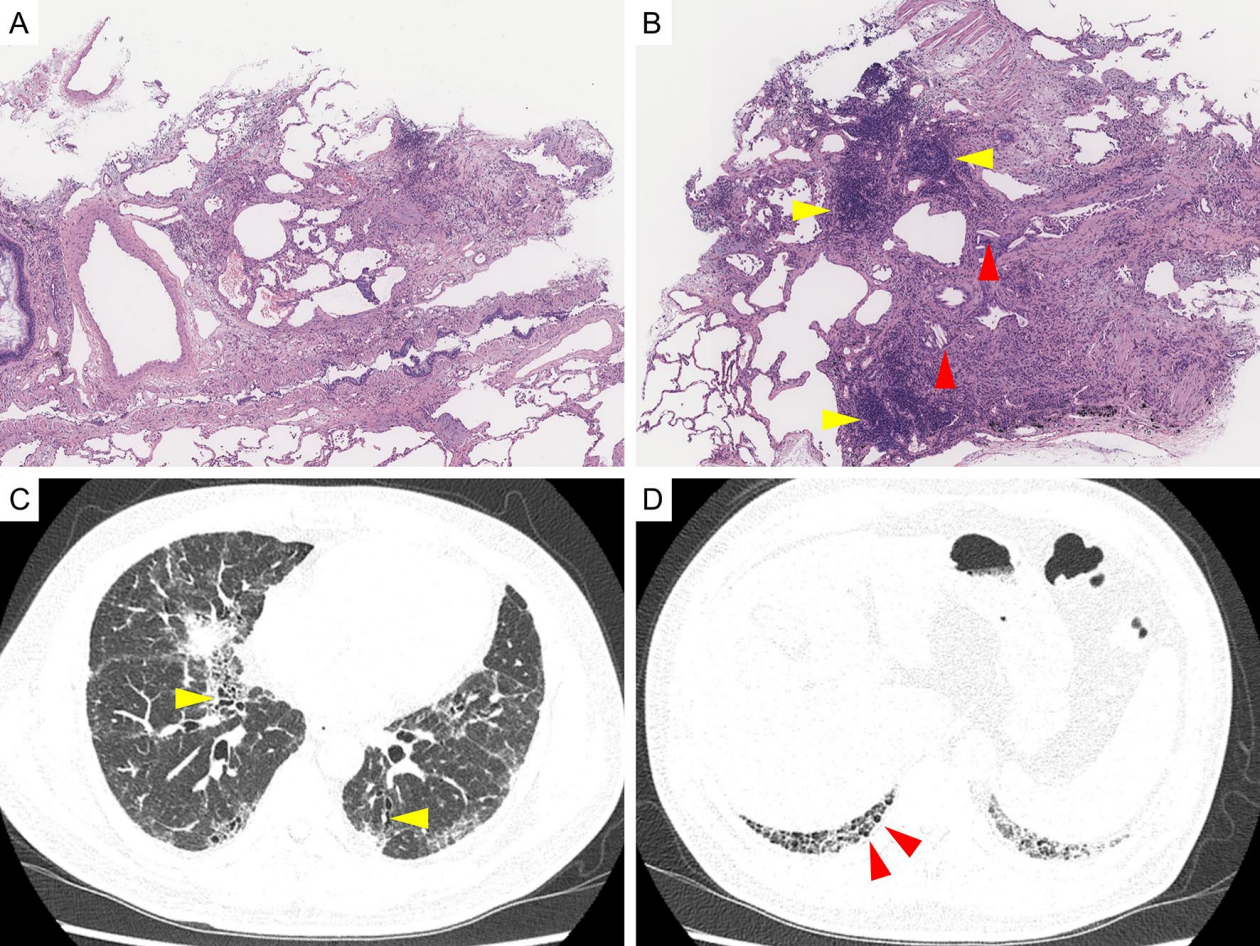
Another case in which the pathology diagnosis by TBLC was inconsistent with the diagnosis of MDD. (**A**), (**B**): pathology by TBLC. Dense fibrosis were observed. In addition, lymphoid follicles with germinalcenter (orange arrow) and giant cells with cholesterol clefts (red arrow) were observed. (**C**),(**D**): HRCT. Traction bronchiectasis (yellow arrow) and honeycombing (red arrow) were observed. But small centrilobular nodules or other findings suggestive of connective tissue disease associated interstitial lung diesase were not observed.

In the second case (Fig. 3), pathology showed patchy interstitial dense fibrosis, as well as a strong lymphocyte infiltrate and the formation of lymph follicles with germinal centers (Fig. 3A,B). Although multinucleated giant cells with cholesterol clefts were observed in some places, no granulomas or enhanced inflammation in the centrilobules were observed. We determined that the basic histological pattern of this case was UIP, but the pathological diagnosis was highly suspicious of CTD-ILD, especially RA-ILD. However, HRCT showed mainly findings strongly suggestive of a UIP pattern, such as traction bronchiectasis and reticular shadows, without small centrilobular nodules or rheumatoid nodules (Fig. 3C,D). The clinical information of this patient showed no joint pain and serological abnormalities suggestive of rheumatoid arthritis, such as rheumatoid factor or anti-cyclic citrullinated peptide antibodies. Finally, we diagnosed this patient with IPF pathologically including clinical/radiological infomation, because the patient was not definitively diagnosed with rheumatoid arthritis. However, considering the possibility of rheumatoid arthritis or other connective tissue diseases which may manifest in the future, our approach in this case is to continue to carefully monitor the patient's joint symptoms and follow-up with MDD.

## Discussion

Our study showed that in comparison to MDD diagnosis, the sensitivity of pathological diagnosis alone using TBLC was lower in iNSIP, CTD-ILD, and fibrotic HP compared to IPF. TBLC is known to be effective for diagnosing ILDs, but is not always concordant with SLB^7,8^. We aimed to identify which ILDs are more sensitive for diagnosis using TBLC, as this information is lackingin existing literature.

In the COLDICE study^8^, pathological diagnoses using TBLC and SLB were concordant in 69.2% cases. For cases with discordant diagnosis, the pathological diagnosis of SLB and TBLC were divided into UIP-IPF and HP^9^. This suggests that diagnosis using TBLC may be less sensitive in HP, especially fibrotic HP. Romagnoli et al. also reported a low concordance rate (38%) between the TBLC and SLB^7^. Their study included 9 cases of IPF;there were 4 cases (44%) where both TBLC and SLB were used to diagnose UIP-IPF. Only 2 cases of the 12 non-IPF cases (17%) showed concordance between the two methods for the MDD and pathological diagnoses^7^, suggesting that the diagnostic sensitivity of TBLC may be lower in non-IPF diseases. These findings corroborate the hypotheses of previous studies.

In this study, we were able to collect pathological diagnoses performed based on the limited clinical information available in clinical practice, as well as pathological diagnoses performed with reference to all clinical/radiological information referenced in the MDD at some institutions, and compare these two groups. If clinical/radiological information is available, pathologists can access the information about problems during tissue sampling and/or confirmed collagen diseases, improving diagnostic accuracy. The addition of clinical/radiological information resulted in a 15% improvement in the sensitivity of pathological diagnosis using TBLC. As previously reported, pathological findings aside from the UIP pattern may be difficult to distinguish using TBLC^10^. The difference in the ability to recognize the pathology on TBLC reported in the above study could be a reason for the difference in diagnostic sensitivity between IPF and other ILDs. Moreover, it is important that in the clinical/radiological information, the TBLC specimen contains all pathological abnormalities of the patients for an accurate diagnosis. For instance, in this study, the diagnosis of iNSIP was challenging even with available clinico-radiological information. Bondue et al. reported that in 16 cases diagnosed with NSIP pattern using TBLC, only one case was also diagnosed with NSIP pattern using SLB^14^. This is owing to the difficulty in evaluating NSIP patterns using TBLC, where only a limited size of the specimen can be observed. When having access to clinical/radiological information, the pathologist is more equipped to identify potential sampling issues with the submitted histopathology specimen (e.g. mis-sampling or severe crush artifact) and whether to consider certain subtle histological findings as diagnostically sufficient. Particularly in diseases other than IPF, considering whether subtle histological findings are consistent with the HRCT pattern or the clinically suspected disease can lead to an accurate diagnosis. As a result, the sensitivity of pathological diagnosis is presumably increased when clinical/radiological information is accessible.

We compared the pathological and MDD diagnosis using TBLC in a large multi-institutional series. A major limitation was the lack of a comparative study of the pathological diagnosis using TBLC with the MDD diagnosis using SLB, the current gold standard for diagnosis of ILD^2^. Furthermore, sensitivity values obtained here are not synonymous with the true diagnostic sensitivity of TBLC matched against MDD-SLB. However, our main purpose was to examine which ILD categories can be diagnosed with high sensitivity using TBLC alone. We concluded that the sensitivity of TBLC is higher in IPF, but lower in iNSIP and ILD with secondary causes, such as HP or CTD-ILD. While our findings are in concordance with previously published small series comparing TBLC and SLB^7–9^, additional studies are required to further support our conclusion.

The current study had the following other limitations. We did not address such variables as number of TBLC specimens collected, the site of sampling, and inter-institutional variability. In addition, this was a retrospective study, and there were no uniform pre-defined criteria for the administration of the TBLC. This study evaluated only the sensitivity of pathology diagnosis using TBLC to look at disease-specific differences in ILD. Specificity or accuracy were not considered in this study. The ontology of pathological diagnosis and differential diagnosis were also not considered in this study.

## Conclusion

The sensitivity of pathological diagnosis with TBLC was lower in iNSIP, CTD-ILD, and fibrotic HP compared to IPF. However, with the addition of all clinical/radiological information, the sensitivity of pathological diagnosis by TBLC has increased and is no less sensitive than IPF for all diseases except iNSIP.

## Data Availability

The datasets used and/or analysed during the current study available from the corresponding author on reasonable request.
